# Inositol Hexakisphosphate Kinase 3 Regulates Metabolism and Lifespan in Mice

**DOI:** 10.1038/srep32072

**Published:** 2016-08-31

**Authors:** Yusuke Moritoh, Masahiro Oka, Yoshitaka Yasuhara, Hiroyuki Hozumi, Kimihiko Iwachidow, Hiromitsu Fuse, Ryuichi Tozawa

**Affiliations:** 1 Cardiovascular and Metabolic Drug Discovery Unit, Pharmaceutical Research Division, Takeda Pharmaceutical Company Limited, Fujisawa, Japan; 2 Integrated Technology Research Laboratories, Pharmaceutical Research Division, Takeda Pharmaceutical Company Limited, Fujisawa, Japan

## Abstract

Inositol hexakisphosphate kinase 3 (IP6K3) generates inositol pyrophosphates, which regulate diverse cellular functions. However, little is known about its own physiological role. Here, we show the roles of IP6K3 in metabolic regulation. We detected high levels of both mouse and human *IP6K3* mRNA in myotubes and muscle tissues. In human myotubes, *IP6K3* was upregulated by dexamethasone treatment, which is known to inhibit glucose metabolism. Furthermore, *Ip6k3* expression was elevated under diabetic, fasting, and disuse conditions in mouse skeletal muscles. *Ip6k3*^−/−^ mice demonstrated lower blood glucose, reduced circulating insulin, deceased fat mass, lower body weight, increased plasma lactate, enhanced glucose tolerance, lower glucose during an insulin tolerance test, and reduced muscle *Pdk4* expression under normal diet conditions. Notably, *Ip6k3* deletion extended animal lifespan with concomitant reduced phosphorylation of S6 ribosomal protein in the heart. In contrast, *Ip6k3*^−/−^ mice showed unchanged skeletal muscle mass and no resistance to the effects of high fat diet. The current observations suggest novel roles of IP6K3 in cellular regulation, which impact metabolic control and lifespan.

Inositol hexakisphosphate kinases (IP6Ks) are involved in diverse cellular signalling^1^,^2^, by generating inositol pyrophosphates and interacting with other cellular components. Inositol pyrophosphates are highly energetic molecules, and the phosphorylation of their already phosphorylated inositol hydroxyl group is catalysed by two different classes of enzymes: IP6Ks with 5-kinase activities^3^,^4^,^5^ and diphosphoinositol pentakisphosphate kinases (PPIP5Ks) with 1-kinase activity^6^,^7^,^8^. The IP6K 5-kinase activity pyrophosphorylates myo-inositol (1,3,4,5,6)-pentakisphosphate (InsP_5_) to 5-PP-InsP_4_, InsP_6_ to 5-PP-InsP_5_ (5-InsP_7_)^9^, and 1-PP-InsP_5_ to 1,5-PP2-InsP_4_ (InsP_8_), respectively. Among these, 5-InsP_7_ is possibly the most studied inositol pyrophosphate generated by IP6Ks, and shows diverse biological effects by target-protein binding and pyrophosphorylation, in which the energetic pyrophosphate group of 5-InsP_7_ donates its β-phosphate to a pre-phosphorylated serine residue of the target protein^10^. Although an additional naturally-occurring isomer of 5PPIP5K-generated InsP_7_ exists wherein a diphosphate group is placed at the 1-carbon (1-InsP_7_), most mammalian cellular InsP_7_ appear to be comprised of the 5-isomer (5-InsP_7_)^3^,^11^.

Mammals have three subtypes of IP6K: IP6K1–3^1^. Recent studies using knockout mice have revealed the physiological importance of the first two IP6Ks in regulating diverse cellular functions. *Ip6k1* gene deletion in mice resulted in growth retardation, reduced levels of circulating insulin from pancreatic beta-cells, and male sterility secondary to defective spermatogenesis^12^. In addition, knockout studies of *Ip6k1* demonstrated its role in regulating platelet polyphosphate levels^13^ and neutrophil function in innate immunity^14^. A recent study also showed enhanced Akt signalling in the liver, fat, and muscle, thereby inducing increased insulin sensitivity wherein IP6K1-generated 5-InsP_7_ bound to the Akt-PH domain and inhibited insulin and insulin-like growth factor 1 (IGF1)-induced phosphatidylinositol (3,4,5)-trisphosphate signalling^15^. In contrast, *Ip6k2* knockout mice did not exhibit growth, fertility, or insulin secretion defects but were resistant to ionizing radiation and their fibroblasts manifested up-regulated DNA repair as well as interferon-β resistance^16^. These mice were also more susceptible to squamous cell carcinoma in the oral cavity and oesophagus compared to wild-type mice when given water containing a carcinogen. Notably, IP6K2, via 5-InsP_7_ synthesis, represents a major mediator of cancer cell migration and tumour metastasis in cell culture and in intact mice^17^. These observations demonstrated that although both generate the same form of 5-InsP_7_, the functional redundancy between IP6K1 and IP6K2 is incomplete.

IP6K3 is *less* well characterized compared to IP6K1 and IP6K2^18^. Northern blotting has shown abundant *Ip6k3* mRNA in the rat cerebellum, and a GFP-IP6K3 fusion protein localized to both the cytoplasm and the nucleus when tested in HEK293 cells^18^. A study focusing on brain function demonstrated that IP6K3 physiologically regulates the morphology and synapse formation of cerebellar Purkinje cells through interacting with other proteins in the mouse brain^19^. Little is known, however, regarding the physiological role of IP6K3 in the whole body. Considering the known importance of the other two IP6K isoforms, we performed *in vitro* and *in vivo* analyses to determine the organismal role of IP6K3. The present study is the first to describe the role of IP6K3 in muscles. Here we show that *Ip6k3* mRNA is highly expressed and responsive to energy and disease status in muscles and that *Ip6k3* gene deletion induces metabolic changes and affects lifespan. We suggest that IP6K3 likely senses the energy status in this tissue and regulates downstream signalling that impacts metabolic control and lifespan in mice.

## Results

### Mouse and human *IP6K3* mRNAs are highly expressed in skeletal muscles and *Ip6k3* is induced by myotube formation in C2C12 cells

**Figure 1:**
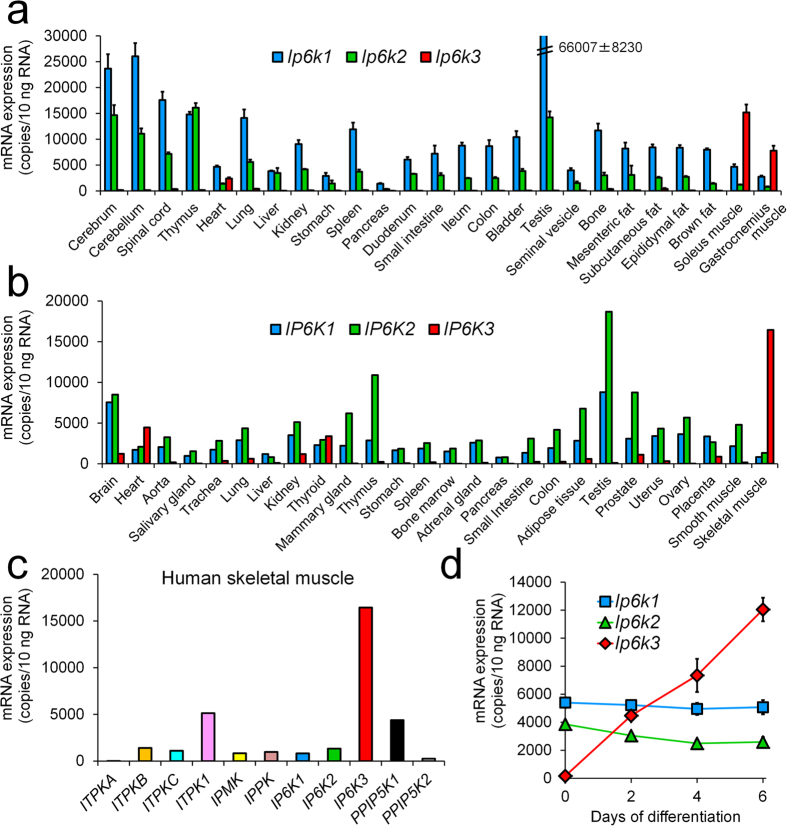
Mouse *Ip6k3* and human *IP6K3* mRNA are highly expressed in skeletal muscles, and *Ip6k3* is induced by myotube formation in C2C12 cells. Tissue distribution of each mRNA in C57BL/6J mice (**a**) and humans (**b**). Expression of inositol phosphate kinase family genes in human skeletal muscle tissues (**c**). Expression profiles of each mRNA during C2C12 myotube formation (**d**). Mouse *Ip6k3* and human *IP6K3* were highly expressed in skeletal muscles. Among the inositol kinase family members tested, *IP6K3* showed the highest mRNA expression in human skeletal muscle. *Ip6k3* mRNA was induced during myotube formation in C2C12 cells. Means ± SD of four mice consisting of two technical replicates for (**a**), means of two technical replicates for human pooled samples in (**b**,**c**), and means ± SD of three independent samples consisting of two technical replicates for (**d**). *ITPKA-C*, inositol-trisphosphate 3-kinase A-C; *ITPK1*, inositol-tetrakisphosphate 1-kinase; *IPMK*, inositol polyphosphate multikinase; *IPPK*, inositol-pentakisphosphate 2-kinase; *PPIP5K1*&*2*, diphosphoinositol pentakisphosphate kinase 1&2.

We first examined *Ip6k3* mRNA tissue distribution in mice. Absolute mRNA level quantification revealed that *Ip6k3 was* highly expressed in murine skeletal muscles (the soleus and gastrocnemius muscles) followed by the heart (Fig. 1a). *Ip6k3* was also detectable in the brain and other tissues at much lower levels. Similar results were obtained in human tissues (Fig. 1b), which also revealed the thyroid as an *IP6K3*-expressing site. Among the inositol kinases, *IP6K3* showed the highest mRNA expression in human skeletal muscles (Fig. 1c). As IP6K3 reportedly functions in the brain^19^, the present study also characterized *IP6K3* brain expression, revealing brain-region dependent *IP6K3* expression in humans (Fig. S1). In contrast, the reported lack of *Ip6k3* expression in mouse insulinoma MIN6 cells^20^ was reconfirmed in our study. In addition, neither the rat beta cell line INS-1 832/13 nor human islets, which contain non-beta endocrine cells, expressed *IP6K3* (Fig. S2).

We next explored the *Ip6k3* profiles in skeletal muscle cells *in vitro*. Quantitative PCR analysis of mouse C2C12 cells demonstrated that *Ip6k3* mRNA in unfused myoblasts was almost undetectable whereas *Ip6k1* and *Ip6k2* were measurable and expressed equally. However, during myotube formation, *Ip6k3* gene expression was robustly induced whereas *Ip6k1* and *Ip6k2* expression remained unchanged (Fig. 1d). Furthermore, when an AcGFP-human IP6K3 fusion protein construct was transfected into mouse C2C12 cells, the resultant AcGFP-IP6K3 was localized to both the cytoplasm and the nucleus (Fig. S3). Similar images were obtained from the human SJCRH30 rhabdomyosarcoma cell line, which expresses detectable *IP6K3* mRNA baseline levels (Figs S3 and S4).

### *Ip6k3* mRNA expression is induced in diabetic, fasting, and disuse conditions in mouse skeletal muscles

**Figure 2:**
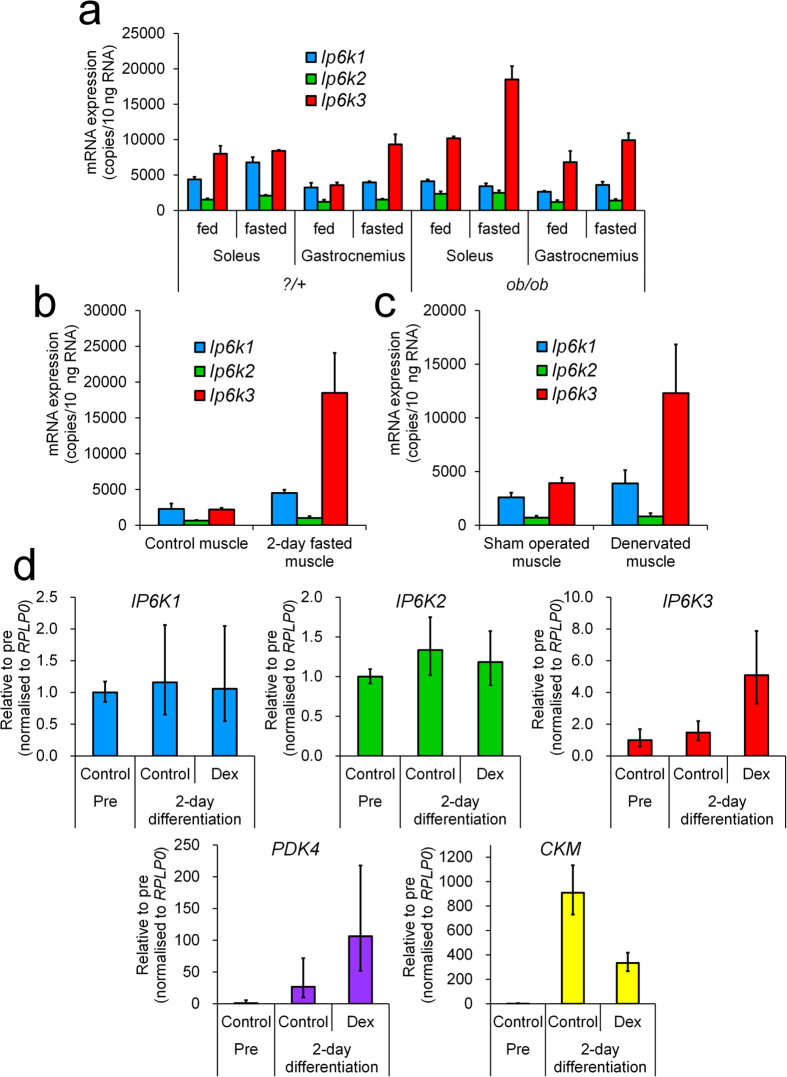
*Ip6k3* mRNA is induced in diabetic, fasting, and disused conditions in mice, and *IP6K3* mRNA is elevated by dexamethasone in human primary myotubes. *Ip6k1*, *Ip6k2*, and *Ip6k3* mRNA expression in the skeletal muscle from ?/+ littermates and *ob/ob* mice (**a**), in the gastrocnemius muscle of 2 days-fasted C57BL/6J mice (**b**), and in the denervated gastrocnemius muscle in C57BL/6J mice (**c**). Human primary skeletal myoblast cells were treated with 2% horse serum containing medium with or without dexamethasone and the respective mRNA levels were measured (**d**). *Ip6k3* expression was induced by fasting and altered in the skeletal muscles of diabetic *ob/ob* mice. In addition, elevation of *Ip6k3* mRNA was observed in 2 day-fasted gastrocnemius muscles and denervated gastrocnemius muscles at 2 days after operation. When human primary myotubes were treated with dexamethasone, which inhibits glucose metabolism, *IP6K3* mRNA expression was elevated. Means ± SD of three ?/+ and *ob/ob* mice consisting of two technical replicates for (**a**), means ± SD of three mice consisting of two technical replicates for (**b**), means ± SD of six sham-operated and denervated mice consisting of two technical replicates for (**c**), means and range of three independent samples consisting of two technical replicates for (**d**). For (**d**), the comparative C_T_ method was used and the ranges given were determined by evaluating the expression: 2^−ΔΔCT^ with ΔΔC_T_ + s and ΔΔC_T_ − s, where s = the standard deviation of the ΔΔC_T_ value. Dex, dexamethasone.

We investigated *Ip6k3* expression changes in food and muscle-related disease conditions such as diabetes and muscle-disuse atrophy. In normal mice, fasting conditions increased *Ip6k3* expression by 1.1- and 2.6-fold in the soleus and gastrocnemius muscles, respectively (Fig. 2a). In addition, *Ip6k3* expression was elevated in the soleus and gastrocnemius muscles of non-fasting diabetic *ob/ob* mice by 1.3- and 1.9-fold, respectively, compared to normal mice (Fig. 2a); fasting further elevated this *Ip6k3* expression by 2.2- and 1.1-fold (Fig. 2a). A longer 2-day fasting period increased *Ip6k3* expression by 8.4-fold in the gastrocnemius muscle in C57BL/6J mice compared to the non-fasting condition (Fig. 2b). In addition, denervated gastrocnemius muscle (2 days after operation), which experiences impaired glucose metabolism and muscle atrophy, showed 3.1-fold increased *Ip6k3* expression (Fig. 2c).

### *IP6K3* mRNA is induced in dexamethasone-treated human primary myotubes

We tested the effect of dexamethasone, a synthetic glucocorticoid that reportedly inhibits glucose metabolism in skeletal muscles^21^, in human primary myotubes. Myotube formation was observed following two days culture with medium containing 2% horse serum. Conversely, dexamethasone treatment resulted in inhibited myotube formation, attenuated *CKM* expression, and a 4.0-fold increase in pyruvate dehydrogenase kinase 4 (*PDK4*) mRNA expression, which inhibits glucose utilization and facilitates fat oxidation^22^. As observed with *PDK4*, *IP6K3* expression was elevated 3.5-fold by dexamethasone treatment, whereas *IP6K1* and *IP6K2* expression was unchanged (Fig. 2d).

### General observations of *Ip6k3* ^−/−^ mice

As described in the Methods section, B6;129-*Ip6k3*^−/−^ mice, B6.Cg-*Ip6k3*^−/−^ mice, and their respective wild-type littermates were used in the present study. A test of five mating pairs (male *Ip6k3*^−/−^ and female *Ip6k3*^−/−^ mice, B6;129-*Ip6k3*^−/−^ mice) resulted in 100% pregnancy rate and five females gave birth to 28 pups (14 males and 14 females), demonstrating normal fertility.

### Food consumption, body composition, and blood parameters of *Ip6k3* ^−/−^ mice

**Figure 3:**
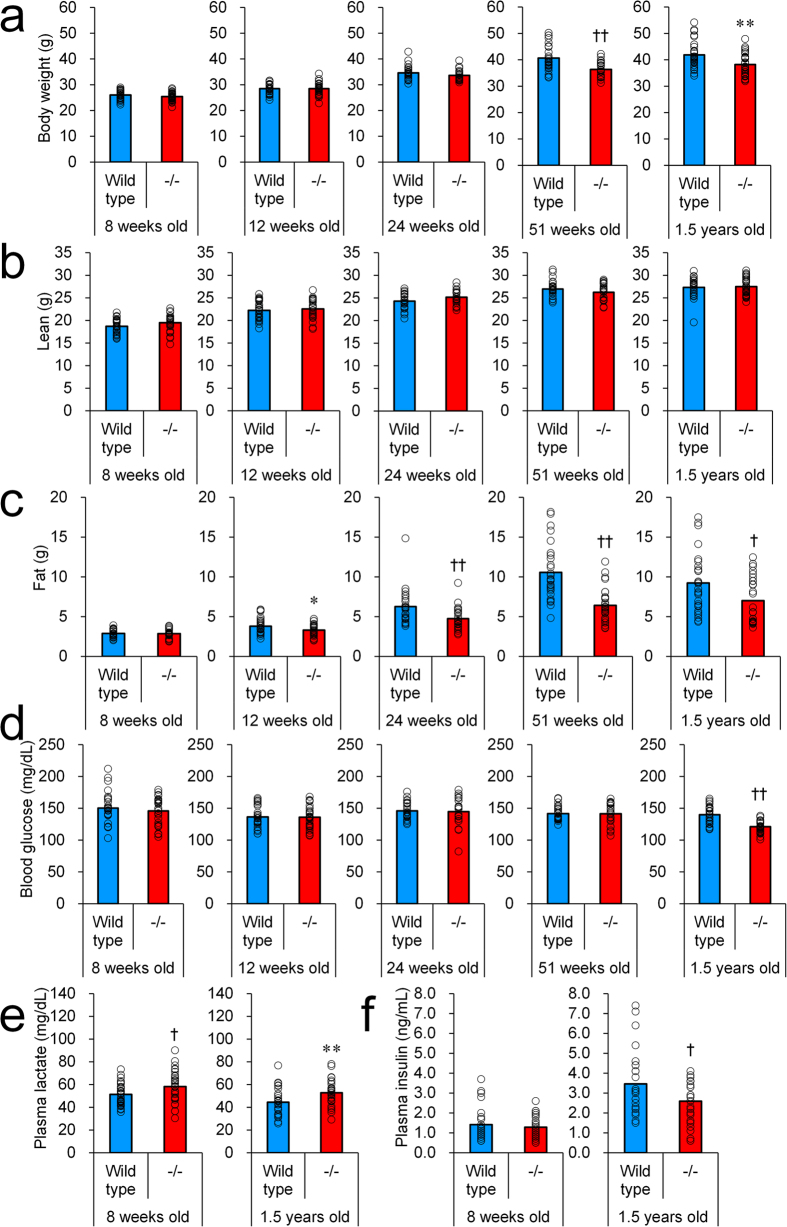
Lower body weight, reduced fat mass, decreased blood glucose, higher plasma lactate, and reduced insulin in *Ip6k3*^−/−^ mice. Body weight (**a**), lean mass (**b**), fat mass (**c**), blood glucose (**d**), plasma lactate (**e**), and plasma insulin (**f**) of *Ip6k3*^−/−^ mice and their wild-type littermates at the indicated ages. The observations became evident with ageing. B6.Cg-*Ip6k3*^−/−^ and wild-type littermates were used for the analysis. The mean values and individual values are shown. *P < 0.05 and **P < 0.01 by the Student’s *t*-test, and ^†^*P* < 0.05 and ^††^*P* < 0.01 by the Aspin-Welch test. n = 30 for 8 and 12 weeks old wild-type and *Ip6k3*^−/−^ mice, n = 24 for 24 weeks old wild-type and *Ip6k3*^−/−^ mice, n = 27 and 28 for 51 weeks old wild-type and *Ip6k3*^−/−^ mice, n = 28 and 29 for 1.5 years old wild-type and *Ip6k3*^−/−^ mice.

B6.Cg-*Ip6k3*^−/−^ mice and their wild-type littermates were utilized in the present analysis. Wild-type and *Ip6k3*^−/−^ mice showed similar levels of food intake (Table 1). Although body weight was unchanged in *Ip6k3*^−/−^ compared to wild-type mice at 8 to 24 weeks of age, those of *Ip6k3*^−/−^ mice were lower from 51 weeks to 1.5 years of age (9–11%) (Fig. 3a). Body composition of *Ip6k3*^−/−^ mice determined using Echo-MRI (Fig. 3b,c) revealed unchanged lean mass and decreased fat mass (−13 to −39%) from 12 weeks to 1.5 years of age. *Ip6k3*^−/−^ mice showed decreased levels of non-fasting blood glucose from 1.5 years of age (Fig. 3d). In contrast, plasma lactate levels were increased at 8 weeks and 1.5 years of age by 1.1–1.2-fold (Fig. 3e). Plasma insulin levels were unchanged at 8 weeks of age, whereas they were decreased by 25% in *Ip6k3*^−/−^ mice at 1.5 years of age (Fig. 3f).

### Lower glucose levels upon glucose and insulin tolerance testing in *Ip6k3* ^−/−^ mice

**Figure 4:**
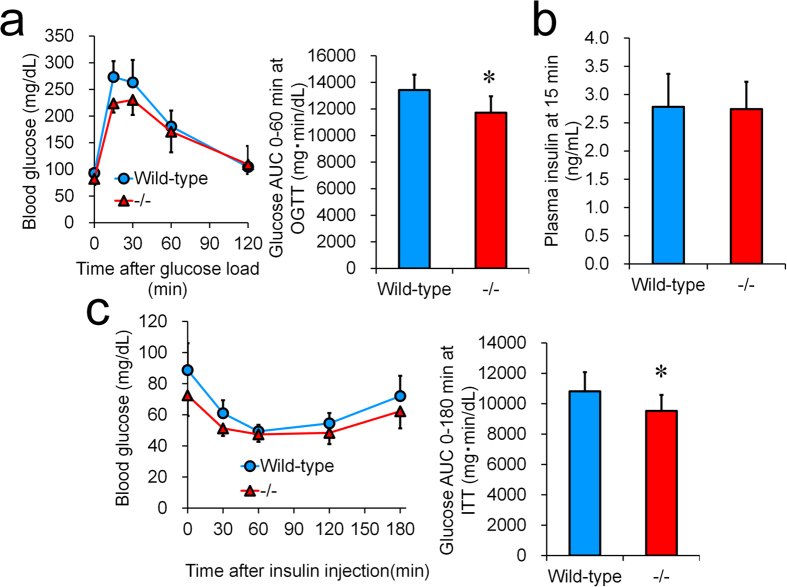
Lower glucose levels in glucose and insulin tolerance testing of *Ip6k3*^−/−^ mice. An oral glucose tolerance test was performed in 15 weeks old mice (**a**). Plasma insulin levels at 15 min after glucose loading are shown (**b**). Insulin (0.75 U/kg) was injected to 12 weeks old mice (**c**). B6;129-*Ip6k3*^−/−^ and wild-type littermates were used for the analysis. Values represent the means ± SD. n = 8. *P < 0.05 by the Student’s *t*-test.

Glucose tolerance was investigated by applying an oral glucose tolerance test in B6;129-*Ip6k3*^−/−^ mice and their wild-type littermates (Fig. 4a). After oral gavage of glucose (2 g/kg), *Ip6k3*^−/−^ mice showed significant improvements in glucose tolerance (Fig. 4a). Plasma insulin levels at 15 min after an oral glucose load were unchanged (Fig. 4b). As observed for the oral glucose tolerance test, B6;129-*Ip6k3*^−/−^ mice showed lower glucose levels compared to their wild-type littermates during an insulin tolerance test (Fig. 4c).

### Prolonged lifespan of *Ip6k3* ^−/−^ mice

**Figure 5:**
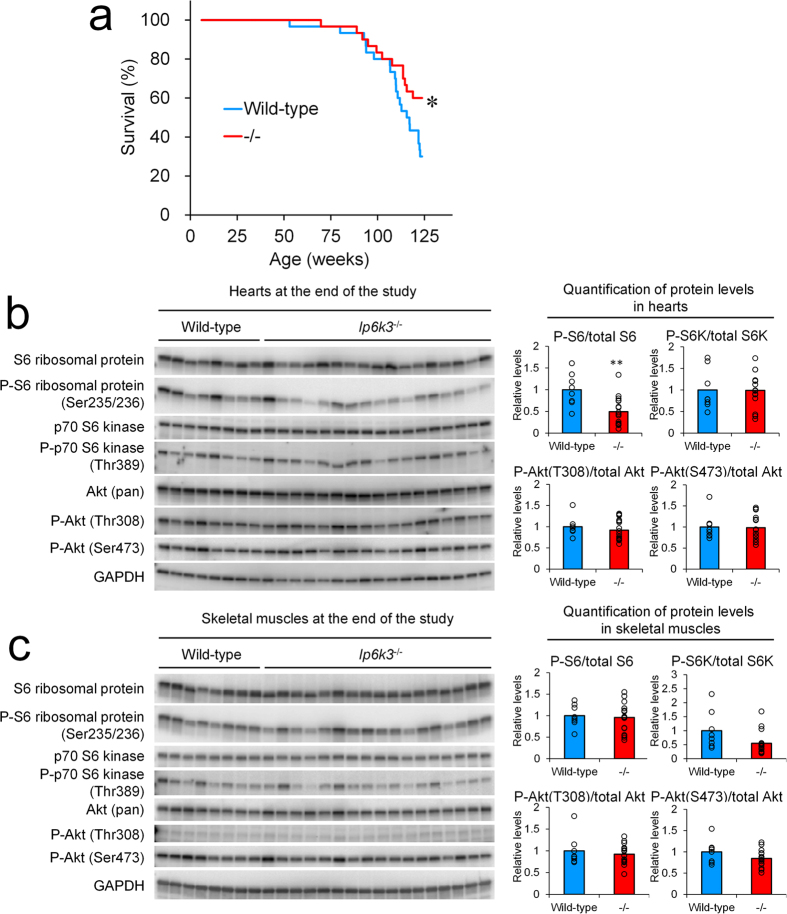
Extended lifespan of *Ip6k3*^−/−^ mice. Kaplan-Meier survival curves show a significant (log-rank χ^2^ = 4.2587 *P < 0.05) lifespan extension in *Ip6k3*^−/−^ mice ((**a**) n = 30 for each group). Western blot analysis of signalling proteins and quantitation of proteins in the hearts and the skeletal muscles at the end of the lifespan study (**b,c**). Decreased phospho-S6 ribosomal protein was observed in the heart. B6.Cg-*Ip6k3*^−/−^ and wild-type littermates were used for the analysis. The mean values and individual values are shown. **P < 0.01 by the Student’s *t*-test

Considering the roles of IP6Ks in regulating diverse cell signalling such as for metabolism control, we were interested to explore the possible association of IP6K3 with lifespan. As shown in Fig. 5a, the lifespan of *Ip6k3*^−/−^ mice (B6.Cg-*Ip6k3*^−/−^) was significantly extended compared to wild-type littermates. The observed causes of death were general factors such as lymphoma and liver tumour, and we observed no differences in tumour formation in skeletal muscles or elsewhere in the *Ip6k3*^−/−^ mice. As *Ip6k3* is not expressed throughout the body but shows relatively restricted expression in the muscles, it was further of interest to explore its role in molecular signalling. We observed decreased phospho-S6 ribosomal protein in the heart of *Ip6k3*^−/−^ mice at the end of the study period independent of the phosphorylation status of the upstream molecules S6 kinase and AKT^23^ (Fig. 5b). In contrast, decreased phospho-S6 ribosomal protein was not observed in the skeletal muscles (Fig. 5c). Although the phosphorylation status of S6K and AKT of skeletal muscles in *Ip6k3*^−/−^ mice tended to be reduced, these changes did not reach statistical significance.

### Similar levels of blood parameters and body weight in high fat diet fed *Ip6k3* ^−/−^ compared to wild-type mice

**Figure 6:**
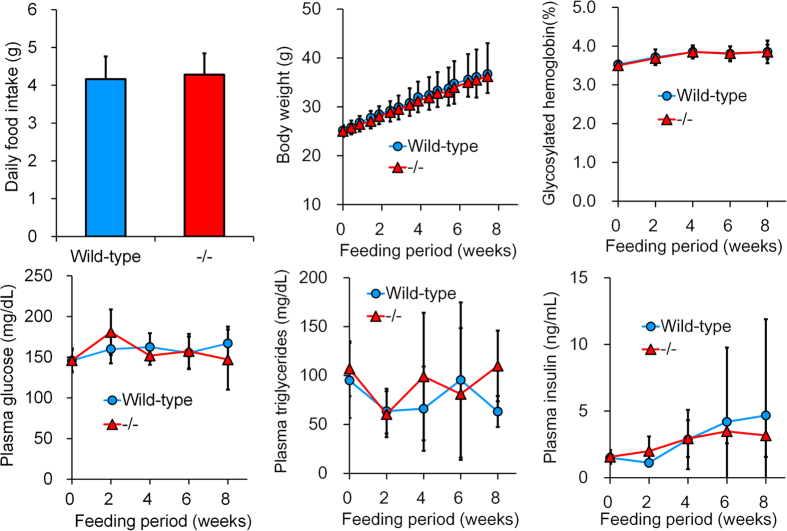
*Ip6k3*^−/−^ mice show no resistance to high-fat diet feeding. *Ip6k3*^−/−^ mice were fed a high fat diet (45% kcal as fat) from 7 to 15 weeks old. *Ip6k3*^−/−^ mice showed similar levels of the indicated parameters compared to wild-type littermates during the 8-week high fat diet feeding period. B6;129-*Ip6k3*^−/−^ and wild-type littermates were used for the analysis. Values represent the means ± SD. n = 8.

The impact of *Ip6k3* deletion was investigated under a high fat diet condition (B6;129-*Ip6k3*^−/−^ mice). However, we observed no changes in food intake, body weight, plasma glucose, triglycerides, or insulin levels in *Ip6k3*^−/−^ mice compared to wild-type littermates during the 8 weeks in which mice were fed the high fat D12451 diet (Fig. 6).

### No impact on basal skeletal muscle mass and denervation-induced skeletal muscle atrophy but reduced levels of *Pdk4* in *Ip6k3* ^−/−^ mice

**Figure 7:**
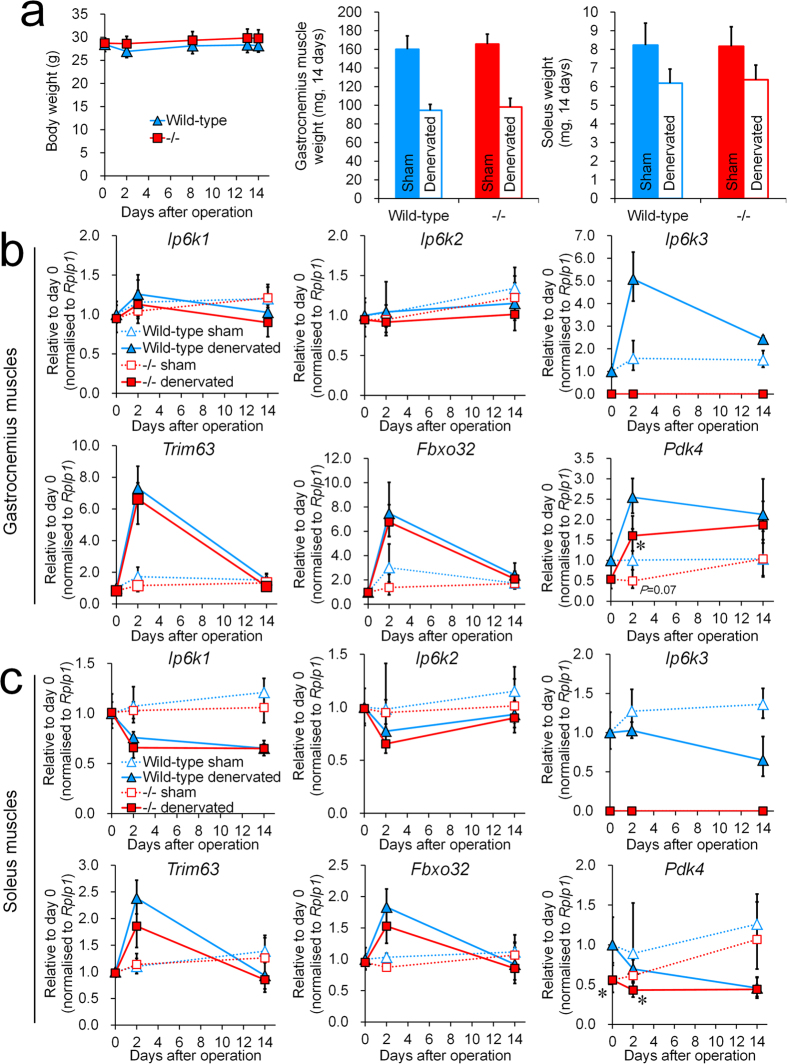
*Ip6k3*^−/−^ mice have no impact on basal skeletal muscle mass and denervation-induced skeletal muscle atrophy but exhibit reduced levels of *Pdk4*. Body, gastrocnemius muscle, and soleus weights (**a**), and gene expression in the gastrocnemius muscles (**b**) and soleus (**c**) after the denervation operation. *Ip6k3* deletion had no impact on basal muscle mass and denervation-induced muscle loss, which was measured 14 days following the operation. *Pdk4* was down-regulated in the muscles of *Ip6k3*^−/−^ mice. B6;129-*Ip6k3*^−/−^ and wild-type littermates were used for the analysis. *P < 0.05 by the Student’s *t*-test. Means ± SD of 6 mice for (**a**). Means and range are shown for (**b**,**c**). n = 4 and 6 for wild-type and *Ip6k3*^−/−^ mice at day 0, n = 4 and 5 for wild-type and *Ip6k3*^−/−^ mice at day 2, n = 6 for wild-type and *Ip6k3*^−/−^ mice at day 14. For calculating gene expression in (**b**,**c**), the comparative C_T_ method was used and the ranges given were determined by evaluating the expression: 2^−ΔΔCT^ with ΔΔC_T_ + s and ΔΔC_T_ − s, where s = the standard deviation of the ΔΔC_T_ value. Two technical replicates were performed per sample.

The impact of *Ip6k3* deletion on basal skeletal muscle mass and muscle denervation was investigated using B6;129-*Ip6k3*^−/−^ mice and their wild-type littermates. Body weight was unchanged in *Ip6k3*^−/−^ mice during the study (Fig. 7a); the basal gastrocnemius muscle and soleus weights were unchanged as well (sham-operated muscles). After 14 days following the denervation operation, muscle weight was decreased by 41% and 22% compared to that following sham-operation in the gastrocnemius muscles and soleus in wild-type mice (Fig. 7a). Under this condition, similar decreases were observed in *Ip6k3*^−/−^ mice as well (Fig. 7a). However, mRNA levels quantification showed that *Ip6k3* mRNA was temporally induced at 2 days after the operation and then decreased in the gastrocnemius muscles but not in the soleus (Fig. 7b,c). *Trim63* and *Fbxo32*, E3 ligases that ubiquitinate and degrade target proteins^24^, were transiently induced in both muscle types, and *Ip6k3* deletion had no impact on their mRNA expression. In addition to facilitating muscle degradation, muscle denervation is known to induce metabolic disturbances in a fibre-type dependent manner^25^. *Pdk4*, which switches tissue from glucose catabolism to fatty acid utilization, was induced in denervated gastrocnemius muscles but not in the soleus (Fig. 7b,c), indicating separate conditions of metabolic disturbance between these muscles. Notably, we detected a down-regulation of *Pdk4* mRNA levels in both muscles in *Ip6k3*^−/−^ mice (Fig. 7b,c).

## Discussion

There has been little research performed to elucidate the function of IP6K3 relative to IP6K1 and IP6K2. Here, we first confirmed that *Ip6k3* was highly expressed in muscles. Muscle *Ip6k3* expression was induced by diabetes, fasting, and disuse muscle conditions in mice. In addition, human primary myotubes treated with dexamethasone, which inhibits glucose metabolism, showed *IP6K3* induction. *Ip6k3*^−/−^ mice exhibited reduced glucose, decreased insulin, reduced fat mass, lower body weight, increased lactate, enhanced glucose tolerance, lower glucose during insulin tolerance testing, and longer lifespan compared to wild-type controls. However, in contrast to the changes in metabolism, no impact was observed on basal skeletal muscle mass and the skeletal muscle atrophy induced by denervation. Thus, the present study is the first to demonstrate novel roles of IP6K3 in regulating metabolism and lifespan.

*Ip6k3* expression has been reported to be abundant in the rat brain^18^. Our detailed gene expression analysis revealed that mouse and human *IP6K3* are comparably highly expressed in skeletal muscles, followed by the heart. Notably, *IP6K3* levels were higher than those of any other inositol phosphate kinase in human skeletal muscles. In addition, *Ip6k3* expression, which was undetected in C2C12 myoblasts, robustly increased during myotube differentiation. In contrast, generally available non-muscle cell lines such as HeLa and HEK293 cells did not express *IP6K3* (Fig. S5). These expression profiles indicate that IP6K3 likely has a pivotal role in mature muscles. When tested under fasting conditions, muscle *Ip6k3* was induced and higher *Ip6k3* levels were observed in muscles obtained from diabetic *ob/ob* mice. In addition, denervation-induced disused muscles, which exhibit metabolic disturbance, also showed rapid *Ip6k3* expression induction. Furthermore, dexamethasone inhibition of glucose metabolism also elevated *IP6K3* mRNA in human myotubes. These data indicate that *Ip6k3* levels might be controlled by energy and disease status in skeletal muscles.

*Ip6k1*^−/−^ and *Ip6k2*^−/−^ mice reportedly exhibit normal blood glucose levels under regular diet conditions^12^,^16^. In the present study, *Ip6k3* gene deletion revealed its role in controlling glucose, insulin, fat mass, body weight, and lactate levels in mice. Enhanced glucose tolerance indicates a higher level of glucose disposal in the *Ip6k3*^−/−^ mice. Furthermore, during an insulin tolerance test, glucose levels were lower in *Ip6k3*^−/−^ mice compared to wild-type littermates. Considering the selective and elevated expression of *Ip6k3* in muscle, this tissue is likely to represent the primary tissue responsible for the observed changes in the *Ip6k3*^−/−^ mice. Taking into account that the glycolysis pathway is activated in *Ip6k1*^−/−^ embryonic fibroblasts and in yeast and fungi mutated for *KCS1*, the IP6K ortholog^26^,^27^, *Ip6k3* deletion might have resulted in increased glycolytic activity thereby increasing glucose uptake in muscles. Thus, the increased plasma lactate levels observed in *Ip6k3*^−/−^ mice might be related to increased glycolytic activity. Notably, in the current study *Ip6k3*^−/−^ mice showed no resistance to fat gain induced by 8 weeks of feeding with a high fat diet, in contrast to a previous IP6K1 study^15^. *Ip6k1* is expressed in fat tissue and Chakraborty *et al*. revealed that the 5-InsP_7_ generated by IP6K1 interferes with Akt signalling, leading to insulin resistance and weight gain^15^. In addition, Szijgyarto *et al*. observed that yeasts devoid of *KCS1* exhibit dysfunctional mitochondria, which are required for fatty acid synthesis^27^. These findings indicate that the *Ip6k1* expressed in fat might have a direct role in fat accumulation via mitochondrial function. Thus, the absence of *Ip6k3* expression in fat tissue might underlie the lack of impact observed on fat accumulation in *Ip6k3*^−/−^ mice under a high fat diet condition. Furthermore, the decreased fat mass observed in *Ip6k3*^−/−^ mice under regular diet conditions is likely to be secondary to the improved metabolic parameters.

The *Ip6k3*^−/−^ mice exhibited prolonged lifespan under regular diet conditions, an intriguing effect as *Ip6k3* is not expressed throughout the body but shows relatively restricted expression in the muscles. Furthermore, we observed decreased phospho-S6 ribosomal protein in the heart but not in the skeletal muscles of *Ip6k3*^−/−^ mice at the end of the study period independent of the phosphorylation status of the upstream molecules S6 kinase and AKT, indicating the tissue dependent role of IP6K3. Recently, molecules affecting protein synthesis such as mTOR and S6K1 have been shown to regulate lifespan in mice^28^,^29^, and inhibiting protein synthesis pathways in mice has been demonstrated to prolong lifespan. In addition, a yeast *KCS1* mutant was demonstrated to exhibit reduced protein synthesis, which is regulated by KCS1-produced inositol pyrophosphate^30^. Considering that S6 ribosomal protein phosphorylation physiologically regulates global protein synthesis^31^, this pathway might be related to the extended lifespan of *Ip6k3*^−/−^ mice. Furthermore, *Ip6k3* deletion may directly affect cardiac metabolism through reducing heart *Pdk4* expression (Fig. S6); these hypotheses require further investigation.

A pathway governing protein degradation, mediated by the ubiquitin proteasome system, is activated in atrophying muscle through activation of the E3 ubiquitin ligases *Trim63* and *Fbxo32*^32^,^33^. In the present study, hind limb denervation resulted in muscle mass reduction in the gastrocnemius and soleus muscles, wherein *Trim63* and *Fbxo32* were upregulated. Although *Ip6k3* expression was induced in the gastrocnemius muscles, *Ip6k3* deletion showed no impact on denervation-induced muscle atrophy. Considering that no change was observed in basal muscle weight, *Ip6k3* is unlikely to control muscle mass and demonstrated no significant connection with the ubiquitin proteasome system, at least under the current tested conditions. In comparison, a previous study reported increased S6 ribosomal protein phosphorylation and tissue mass in gastrocnemius muscles in *Ip6k1*^−/−^ mice under basal conditions^15^, which differs from the observations in *Ip6k3*^−/−^ mice that showed unchanged S6 ribosomal protein phosphorylation and tissue mass in skeletal muscles. Considering, however, that reduced S6 ribosomal protein phosphorylation was observed in the heart of *Ip6k3*^−/−^ mice, IP6K1 and IP6K3 might have independent roles in regulating S6 signalling. The development of selective inhibitors of IP6Ks that are effective *in vivo* would likely reveal the role of 5-InsP_7_ in S6 signalling and muscle mass regulation.

The switch from glucose catabolism to fatty acid utilization is regulated by phosphorylation and inactivation of the pyruvate dehydrogenase complex mediated by the kinase PDK4^34^. Here, we found that *Pdk4* baseline expression was decreased in the skeletal muscles and heart of *Ip6k3*^−/−^ mice, indicating a metabolic shift toward glucose oxidation. Notably, *Ip6k3* was upregulated under the conditions wherein *Pdk4* was upregulated (human myotubes and the denervation study). Thus, there might be a biological connection between IP6K3 and PDK4 in muscles.

IP6K1 regulates insulin secretion as evidenced by the decreased serum insulin observed in *Ip6k1*^−/−^mice^12^ and the dysregulated insulin secretion in *Ip6k1* siRNA-treated pancreatic beta cells^20^. We confirmed that *Ip6k3* was undetectable in rodent pancreatic beta cell lines and that no *IP6K3* was observable in human islets, indicating no direct role of IP6K3 in pancreatic endocrine cells. Consistent with this, plasma insulin levels at 15 min after glucose loading was unchanged in mice. However, we observed lowered plasma insulin levels in 1.5 years old *Ip6k3*^−/−^ mice, suggesting that reduced plasma insulin is likely to be secondary to improved metabolic profiles.

It has been suggested that 5-InsP_7_ can act as a proactive metabolic messenger^10^,^35^, and that IP6K3-generated 5-InsP_7_ likely plays a role in regulating downstream signalling. In this study we were not able to determine the *in vivo* levels of 5-InsP_7_ in the muscle of *Ip6k3*^−/−^ mice owing to methodological limitations; however, a new method applicable to tissue samples is under development in our laboratory. In addition, we failed to capture endogenous mouse IP6K3 protein using available antibodies under the current experimental conditions. A recent study has provided evidence that IP6K3 serves as a scaffold to link target proteins, a function not requiring the catalytic activity of IP6K3 demonstrated in mouse brain^19^. Thus, future studies exploring the protein-protein interactions in muscles are essential. Finally, IP6K3 is expressed outside the muscles (*e.g.* in the thyroid) and its function in those tissues needs to be determined.

In conclusion, the present study has revealed that *Ip6k3* is highly expressed in muscles and that its mRNA levels are regulated in response to energy and disease status. The pivotal role of IP6K3 in metabolism and lifespan but not in the control of muscle mass was demonstrated using *Ip6k3*^−/−^ mice. The current observations suggest the existence of novel roles of IP6K3 in sensing energy status and regulating downstream signalling to impact metabolic control and lifespan. Furthermore, the development of a selective inhibitor of IP6K3 might represent a new class of drug to treat diabetes, which might also have the enticing added benefit of potentially extending lifespan.

## Methods

### Reagents and cells

All reagents were purchased from Wako Pure Chemical Industries or Sigma-Aldrich, unless otherwise indicated. C2C12 cells were obtained from the American Type Culture Collection (ATCC). Human primary myoblasts were purchased from Thermo Fisher Scientific.

### Cell culture

C2C12 cells were maintained in Dulbecco’s modified Eagle’s medium containing 25 mM glucose and 10% foetal calf serum (HyClone, GE Healthcare). Differentiation of C2C12 myoblasts was induced by replacing the medium with 2% horse serum (Thermo Fisher Scientific). Cells were cultured in a humidified atmosphere containing 5% CO_2_/95% air at 37 °C.

### Human myotube study

Three independent lots of human skeletal myoblasts were treated with DMEM containing 2% horse serum with or without 1 μM dexamethasone for 2 days, and the collected samples were submitted to quantitative PCR analysis.

### Mice

Male *Lep*^*ob*^*/Lep*^*ob*^ (*ob/ob*; B6.V-*Lep*^*ob*^/J) mice and their non-diabetic, untyped (?/+; B6.V-*Lep*^*ob*^/J) littermates, and male C57BL/6J mice were obtained from Charles River Japan. The fasting duration was 16 hours for *ob/ob* and ?/+ mice, and a longer duration of 2-days was used for C57BL/6J mice. Tissue samples were collected at 8 weeks of age for C57BL/6J mice (for tissue mRNA distribution), 12 weeks of age for ?/+ and *ob/ob* mice, and 9 weeks of age for 2-days fasted C57BL/6J mice and denervated C57BL/6J mice. All animals were housed in cages in a room with controlled temperature (23 °C), humidity (55%), and lighting (lights on from 7:00 am to 7:00 pm) and were maintained on a laboratory chow diet (CE-2, CLEA). The care and use of the animals and the experimental protocols used in this research were approved by the Experimental Animal Care and Use Committee of Takeda Pharmaceutical Company, Ltd. All experiments were performed in accordance with the guidelines and regulations of Takeda Pharmaceutical Company, Ltd. (Shonan Research Center IACUC Guidelines). All animal studies were conducted after a more than 6-day acclimation period. Tissue samples were collected from anaesthetised mice.

#### *Ip6k3* ^−/−^ mice

*Ip6k3*^−/−^ mice lacking from the middle of exon 3 to exon 5 of *Ip6k3* were obtained from Taconic Biosciences (TF1523). This strain lacks the lysine at position at 212 (exon 5) of mouse IP6K3 corresponding to the lysine at position 217 of human IP6K3, which is critical for its enzymatic activity^18^. *Ip6k3* mRNA was undetectable in the muscle of *Ip6k3*^−/−^ mice as determined using quantitative PCR detection of *Ip6k3* sequence at the exon 3–4 boundary. In the present study, we used male *Ip6k3*^−/−^ mice and their wild-type littermates with a mixed genetic background (B6;129-*Ip6k3*^−/−^) [C57BL/6 (75%) and 129SvEv (25%)] and C57BL/6 genetic background mice (B6.Cg-*Ip6k3*^−/−^), which was speed backcrossed five times to C57BL/6 mice.

#### General characterization of *Ip6k3* ^−/−^ mice

Male B6.Cg-*Ip6k3*^−/−^ mice and their wild-type littermates were used for the study. The mice were fed a CE-2 diet. Blood samples were collected at the indicated points from the tail vein (8:00 am, non-fasting condition) for metabolic parameters analysis. Body weight and food consumption were measured at regular intervals, and average daily food consumption per mouse was calculated. Blood glucose levels were measured using a LIFE CHECK (GUNZE). Plasma insulin levels were analysed with an enzyme-linked immunosorbent assay (Millipore). Plasma lactate levels were determined using a commercial kit (Determiner LA; Kyowa Medex). Body composition was quantitated by magnetic resonance imaging to directly measure total body fat mass and total body lean mass on unanaesthetised mice at the indicated ages (EchoMRI-900, Aloka).

### Oral glucose tolerance test and insulin tolerance test

Male B6;129-*Ip6k3*^−/−^ mice and their wild-type littermates at 15 weeks of age (n = 8/group) were fasted overnight and a glucose solution was orally administered (2 g/kg), then blood was collected from the tail vain and the glucose levels were measured by ACCU-CHEK (Roche). Plasma insulin levels were analysed with an enzyme-linked immunosorbent assay (Millipore). For insulin tolerance testing, male B6;129-*Ip6k3*^−/−^ mice at 12 weeks of age (n = 8/group) were fasted overnight and insulin was injected intraperitoneally at 0.75 units/kg (Novo Nordisk), then blood was collected from the tail vain and the glucose levels were measured using LIFE CHECK (GUNZE).

### Estimation of lifespan

Male B6.Cg-*Ip6k3*^−/−^ mice and their wild-type littermates were used. The principal endpoint was the age at death (for mice found dead during daily inspections) or age at euthanasia (for mice deemed unlikely to recover or survive). Mice were examined at least daily for clinical signs, and were euthanized for humane reasons if they were so severely moribund that they were considered, by the researchers and veterinarians, unlikely to survive or recover. A mouse was considered severely moribund if it exhibited more than one of the following clinical signs: (a) rapid weight loss; (b) severe balance or gait disturbance; (c) tumour size over 10% of body weight; or (d) severely ulcerated skin. Tissue samples were collected from anaesthetised mice.

### High fat diet feeding study

In the high fat diet study, male B6;129-*Ip6k3*^−/−^ mice and their wild-type littermates (7 weeks old, n = 8/group) were fed on a standard high-fat diet (D12451, 45% kcal as fat; Research Diets) for 8 weeks, and the body weight and blood parameters were measured using an HLC-723 GHb G8 (Tosoh Bioscience) and a 7180 autoanalyser (Hitachi) at the indicated intervals. Plasma insulin levels were analysed with an enzyme-linked immunosorbent assay (Millipore).

### Muscle denervation

Male B6;129-*Ip6k3*^−/−^ mice and their wild-type littermates at 11 weeks of age were used (n = 4–6/group). Denervation was performed by excising the smaller section of the sciatic nerve (2–3 mm) and the non-denervated hind limb served as a sham-operated control in subsequent analyses, which were carried out 2 and 14 days after the operation. This procedure does not affect the ability of the animal to ambulate. Tissues from mice before the operation were used as pre-control samples. Tissue samples were collected from anaesthetised mice.

### Total RNA and cDNA preparation

Tissues were dissected and stabilized using RNAlater RNA Stabilization Reagent (Thermo Fisher Scientific). Stabilized tissues were homogenised in QIAzol Lysis Reagent, followed by RNeasy kit purification (Qiagen). Cellular RNA samples were prepared using an RNeasy kit. First strand cDNA was synthesized from up to 1.5 μg total RNA per 20 μL reaction using the High-Capacity cDNA Reverse Transcription Kit reverse transcriptase (Thermo Fisher Scientific). Total RNA from various human tissues and brain regions was purchased from TaKaRa Bio Inc. (Premium RNA) and cDNA was synthesized from up to 1 μg total RNA per 20 μL reaction using Superscript II reverse transcriptase (Thermo Fisher Scientific).

### Gene expression analysis

To determine the absolute copy numbers of the target genes, a standard curve was generated by amplifying known concentrations of synthetic oligonucleotides, and the target gene copy number was calculated using this curve. All oligonucleotide primers and dual-labelled (FAM, VIC, HEX - TAMRA, BHQ, MGB) oligonucleotide probes were synthesized by Sigma-Aldrich or Thermo Fisher Scientific (Table S1). The TaqMan gene expression assays used are described in Table S2. Relative mRNA quantification was performed in combination with an internal control gene, *RPLP0* or *Rplp1*. Amplification was performed under the following conditions: initial denaturation at 95 °C for 10 min, followed by 40 cycles of denaturation at 95 °C for 15 s and annealing and extension at 60 °C for 60 s on the ABI PRISM 7900HT Sequence Detector (Thermo Fisher Scientific) using the EXPRESS qPCR supermix (Thermo Fisher Scientific).

### Antibodies

The antibodies used were as follows: phospho-S6 ribosomal protein (Ser235/236) (#4858), S6 ribosomal protein (#2217), phospho-p70 S6 kinase (Thr389) (#9234), p70 S6 kinase (#2708), Akt (pan) (#4691), phospho-Akt (Thr308) (#2965), and phospho-Akt (Ser473) (#4060)(all from CST). Anti-rabbit IgG, HRP-linked antibodies were from CST (#7074) or Jackson Laboratories (111-035-144). GAPDH antibodies were from MBL (M171-7) or CST (#5174).

### SDS-PAGE and western immunoblotting

Sample lysates were prepared by homogenizing in extraction reagent (T-PER, 78510, Thermo Fisher Scientific) with protease (05 892 970 001, Roche) and phosphatase (04 906 837 001; Roche) inhibitor cocktails. Concentration-adjusted protein (Pierce 660 nm Protein Assay) was heated at 100 °C for 5 min in Laemmli sample buffer (Bio-Rad) followed by SDS/PAGE (TGX gel, Bio-Rad). Samples were transferred to a PVDF membrane, blocked in 5% skim milk, and incubated overnight with primary antibodies following by the respective secondary antibodies. Membranes were developed using ECL reagent (RPN2232; GE Healthcare) and immunoreactive bands were quantified with Quantity One Software (Bio-Rad).

### Statistical analyses and calculations

Statistical significance was analysed using the F test, which was used for testing the homogeneity of variances, followed by the Student’s t test (*P* > 0.2 by the F test) or the Aspin-Welch test (*P* ≤ 0.2 by the F test). Student’s t tests and Aspin-Welch tests were conducted at the two-tailed significance levels of 5% (0.05). Cumulative survival curves were constructed using Kaplan-Meier survivorship methods. Differences between the curves were tested for significance by the log-rank test. All data were presented as the means and SD, except for qPCR data generated using the comparative C_T_ (ΔΔC_T_) method (User Bulletin #2, Applied Biosystems). For the comparative C_T_ method, the ΔC_T_ value was determined by subtracting the average internal control C_T_ value from the average target C_T_ value. The calculation of ΔΔC_T_ involves subtraction by the ΔC_T_ calibrator value. The range given for each mRNA level relative to the control was determined by evaluating the expression: 2^−ΔΔCT^ with ΔΔC_T_ + s and ΔΔC_T_ − s, where s = the standard deviation of the ΔΔC_T_ value.

## Additional Information

**How to cite this article**: Moritoh, Y. *et al*. Inositol Hexakisphosphate Kinase 3 Regulates Metabolism and Lifespan in Mice. *Sci. Rep.* **6**, 32072; doi: 10.1038/srep32072 (2016).

## Supplementary Material

Supplementary Information

## Figures and Tables

**Table 1:** Average food intake in *Ip6k3*^−/−^ mice.

	8 weeks old	12 weeks old	24 weeks old	51 weeks old	1.5 years old
Wild-type	3.5 ± 0.1	3.5 ± 0.2	3.6 ± 0.3	3.5 ± 0.2	3.4 ± 0.2
−/−	3.4 ± 0.1	3.6 ± 0.3	3.7 ± 0.1	3.5 ± 0.2	3.4 ± 0.2

B6.Cg-*Ip6k3*^−/−^ and wild-type littermates were used for the analysis. Average daily food consumption per mouse is shown. The means ± SD are given. Cage number = 8 (total 24–30 mice).

## References

[b1] Barker C. J., Illies C., Gaboardi G. C. & Berggren P. O. Inositol pyrophosphates: structure, enzymology and function. Cell. Mol. Life Sci. 66, 3851–3871 (2009). 19714294 10.1007/s00018-009-0115-2 PMC11115731

[b2] Azevedo C., Szijgyarto Z. & Saiardi A. The signaling role of inositol hexakisphosphate kinases (IP6Ks). Adv. Enzym. Regul. 51, 74–82 (2011). 10.1016/j.advenzreg.2010.08.003 21035498

[b3] Lin H. . Structural analysis and detection of biological inositol pyrophosphates reveal that the family of VIP/diphosphoinositol pentakisphosphate kinases are 1/3-kinases. J. Biol. Chem. 284, 1863–1872 (2009). 18981179 10.1074/jbc.M805686200 PMC2615522

[b4] Saiardi A., Erdjument-Bromage H., Snowman A. M., Tempst P. & Snyder S. H. Synthesis of diphosphoinositol pentakisphosphate by a newly identified family of higher inositol polyphosphate kinases. Curr. Biol. 9, 1323–1326 (1999). 10574768 10.1016/s0960-9822(00)80055-x

[b5] Draskovic P. . Inositol hexakisphosphate kinase products contain diphosphate and triphosphate groups. Chem. Biol. 15, 274–286 (2008). 18355727 10.1016/j.chembiol.2008.01.011

[b6] Choi J. H., Williams J., Cho J., Falck J. R. & Shears S. B. Purification, sequencing, and molecular identification of a mammalian PP-InsP5 kinase that is activated when cells are exposed to hyperosmotic stress. J. Biol. Chem. 282, 30763–30775 (2007). 17702752 10.1074/jbc.M704655200 PMC2366029

[b7] Fridy P. C., Otto J. C., Dollins D. E. & York J. D. Cloning and characterization of two human VIP1-like inositol hexakisphosphate and diphosphoinositol pentakisphosphate kinases. J. Biol. Chem. 282, 30754–30762 (2007). 17690096 10.1074/jbc.M704656200

[b8] Wang H., Falck J. R., Hall T. M. & Shears S. B. Structural basis for an inositol pyrophosphate kinase surmounting phosphate crowding. Nat. Chem. Biol. 8, 111–116 (2011). 22119861 10.1038/nchembio.733 PMC3923263

[b9] Shears S. B. Inositol pyrophosphates: why so many phosphates? Adv. Biol. Regul. 57, 203–216 (2015). 25453220 10.1016/j.jbior.2014.09.015 PMC4291286

[b10] Wilson M. S., Livermore T. M. & Saiardi A. Inositol pyrophosphates: between signalling and metabolism. Biochem. J. 452, 369–379 (2013). 23725456 10.1042/BJ20130118

[b11] Albert C. . Biological variability in the structures of diphosphoinositol polyphosphates in Dictyostelium discoideum and mammalian cells. Biochem. J. 327, 553–560 (1997). 9359429 10.1042/bj3270553 PMC1218829

[b12] Bhandari R., Juluri K. R., Resnick A. C. & Snyder S. H. Gene deletion of inositol hexakisphosphate kinase 1 reveals inositol pyrophosphate regulation of insulin secretion, growth, and spermiogenesis. Proc. Natl. Acad. Sci. USA 105, 2349–2353 (2008). 18268345 10.1073/pnas.0712227105 PMC2268139

[b13] Ghosh S. . Inositol hexakisphosphate kinase 1 maintains hemostasis in mice by regulating platelet polyphosphate levels. Blood 122, 1478–1486 (2013). 23782934 10.1182/blood-2013-01-481549

[b14] Prasad A. . Inositol hexakisphosphate kinase 1 regulates neutrophil function in innate immunity by inhibiting phosphatidylinositol-(3,4,5)-trisphosphate signaling. Nat. Immunol. 12, 752–760 (2011). 21685907 10.1038/ni.2052 PMC3140608

[b15] Chakraborty A. . Inositol pyrophosphates inhibit Akt signaling, thereby regulating insulin sensitivity and weight gain. Cell 143, 897–910 (2010). 21145457 10.1016/j.cell.2010.11.032 PMC3052691

[b16] Morrison B. H. . Gene deletion of inositol hexakisphosphate kinase 2 predisposes to aerodigestive tract carcinoma. Oncogene 28, 2383–2392 (2009). 19430495 10.1038/onc.2009.113 PMC3171149

[b17] Rao F. . Inositol pyrophosphates promote tumor growth and metastasis by antagonizing liver kinase B1. Proc. Natl. Acad. Sci. USA 112, 1773–1778 (2015). 25617365 10.1073/pnas.1424642112 PMC4330756

[b18] Saiardi A., Nagata E., Luo H. R., Snowman A. M. & Snyder S. H. Identification and characterization of a novel inositol hexakisphosphate kinase. J. Biol. Chem. 276, 39179–39185 (2001). 10.1074/jbc.M106842200 11502751

[b19] Fu C. . . Inositol hexakisphosphate kinase-3 regulates the morphology and synapse formation of cerebellar purkinje cells via spectrin/adducin. J. Neurosci. 35, 11056–11067 (2015). 26245967 10.1523/JNEUROSCI.1069-15.2015 PMC4524975

[b20] Illies C. . Requirement of inositol pyrophosphates for full exocytotic capacity in pancreatic beta cells. Science 318, 1299–1302 (2007). 18033884 10.1126/science.1146824

[b21] Kuo T., Harris C. A. & Wang J. C. Metabolic functions of glucocorticoid receptor in skeletal muscle. Mol. Cell. Endocrinol. 380, 79–88 (2013). 23523565 10.1016/j.mce.2013.03.003 PMC4893778

[b22] Zhang S., Hulver M. W., McMillan R. P., Cline M. A. & Gilbert E. R. The pivotal role of pyruvate dehydrogenase kinases in metabolic flexibility. Nutr. Metab. (Lond). 11, 10 (2014). 24520982 10.1186/1743-7075-11-10 PMC3925357

[b23] Han E. K. . Akt inhibitor A-443654 induces rapid Akt Ser-473 phosphorylation independent of mTORC1 inhibition. Oncogene 26, 5655–5661 (2007). 17334390 10.1038/sj.onc.1210343

[b24] Bonaldo P. & Sandri M. Cellular and molecular mechanisms of muscle atrophy. Dis. Model. Mech. 6, 25–39 (2013). 23268536 10.1242/dmm.010389 PMC3529336

[b25] Evans O. B. Muscle pyruvate oxidation following denervation and reinnervation. Muscle Nerve 6, 557–560 (1983). 6689053 10.1002/mus.880060804

[b26] Lev S. . Fungal inositol pyrophosphate IP7 is crucial for metabolic adaptation to the host environment and pathogenicity. MBio 6, e00531–15 (2015). 26037119 10.1128/mBio.00531-15 PMC4453010

[b27] Szijgyarto Z., Garedew A., Azevedo C. & Saiardi A. Influence of inositol pyrophosphates on cellular energy dynamics. Science 334, 802–805 (2011). 22076377 10.1126/science.1211908

[b28] Harrison D. E. . Rapamycin fed late in life extends lifespan in genetically heterogeneous mice. Nature 460, 392–395 (2009). 19587680 10.1038/nature08221 PMC2786175

[b29] Selman C. . Ribosomal protein S6 kinase 1 signaling regulates mammalian life span. Science 326, 140–144 (2009). 19797661 10.1126/science.1177221 PMC4954603

[b30] Thota S. G., Unnikannan C. P., Thampatty S. R., Manorama R. & Bhandari R. Inositol pyrophosphates regulate RNA polymerase I-mediated rRNA transcription in Saccharomyces cerevisiae. Biochem. J. 466, 105–114 (2015). 25423617 10.1042/BJ20140798 PMC4325516

[b31] Meyuhas O. Ribosomal protein S6 phosphorylation: four decades of research. Int. Rev. Cell Mol. Biol. 320, 41–73 (2015). 26614871 10.1016/bs.ircmb.2015.07.006

[b32] Gomes M. D., Lecker S. H., Jagoe R. T., Navon A. & Goldberg A. L. Atrogin-1, a muscle-specific F-box protein highly expressed during muscle atrophy. Proc. Natl. Acad. Sci. USA 98, 14440–14445 (2001). 11717410 10.1073/pnas.251541198 PMC64700

[b33] Bodine S. C. . Identification of ubiquitin ligases required for skeletal muscle atrophy. Science 294, 1704–1708 (2001). 11679633 10.1126/science.1065874

[b34] Kwon H. S. & Harris R. A. Mechanisms responsible for regulation of pyruvate dehydrogenase kinase 4 gene expression. Adv. Enzyme Regul. 44, 109–121 (2004). 15581486 10.1016/j.advenzreg.2003.11.020

[b35] Shears S. B. Diphosphoinositol polyphosphates: metabolic messengers? Mol. Pharmacol. 76, 236–252 (2009). 19439500 10.1124/mol.109.055897 PMC2713120

