# Supplementary material for: Inositol Hexakisphosphate Kinase 3 Regulates Metabolism and Lifespan in Mice
Source: Sci Rep. 2016 Aug 31;6:32072. doi: 10.1038/srep32072 (PMC5006000; doi:10.1038/srep32072)
Supplement: Supplementary Information [file srep32072-s1.pdf]

Supplementary Information

Inositol hexakisphosphate kinase 3 regulates metabolism and lifespan in mice

Yusuke Moritoh[1], Masahiro Oka[1], Yoshitaka Yasuhara[1], Hiroyuki Hozumi[1], Kimihiko Iwachidow[2], Hiromitsu Fuse[2] & Ryuichi Tozawa[1]

[1]Cardiovascular and Metabolic Drug Discovery Unit, Pharmaceutical Research Division, Takeda Pharmaceutical Company Limited, Fujisawa, Japan

[2]Integrated Technology Research Laboratories, Pharmaceutical Research Division, Takeda Pharmaceutical Company Limited, Fujisawa, Japan

Supplementary methods

Cells. Human SJCRH30, HeLa, and HEK293 cells were obtained from the American Type Culture Collection (ATCC). SJCRH30 cells were maintained in RPMI1640 medium containing 25 mM glucose and 10% foetal bovine serum (FBS, HyClone, GE Healthcare). HeLa and HEK293 cells were cultured in Eagle's minimum essential medium containing 10% FBS. MIN6 cells were maintained in Dulbecco's modified Eagle's medium containing 25 mM glucose, 15% FBS, and 55 μM 2-mercaptoethanol. INS-1 832/13 cells were grown in RPMI 1640 medium containing L-glutamine, 1 mM sodium pyruvate, 10 mM HEPES, 10% FBS, and 55 μM 2-mercaptoethanol. All cells were cultured in a humidified atmosphere containing 5% CO_2/95% air at 37°C.

Localization of AcGFP-IP6K proteins. The *AcGFP* gene was fused to the N-terminal region of human *IP6K1*, *IP6K2*, and *IP6K3* coding sequences and subcloned into the pEBMulti-Neo vector (Wako). Transfection was performed using FugeneHD (Roche) or Lipofectamine 3000 (Thermo Fisher Scientific). Each construct was transfected into C2C12 cells and SJCRH30 cells, and cells were cultured for an additional 2-4 days. Images were obtained from a highly sensitive charge-coupled device (CCD) camera fitted to the microscope (DP73/IX71; Olympus).

Human islet cDNA. Human islet cDNA (50 µL cDNA synthesized from 0.5 µg total RNA from each of 3 normal individuals: a 51-year-old male, a 52-year-old female, and a 50-year-old female) was obtained from Cosmobio.

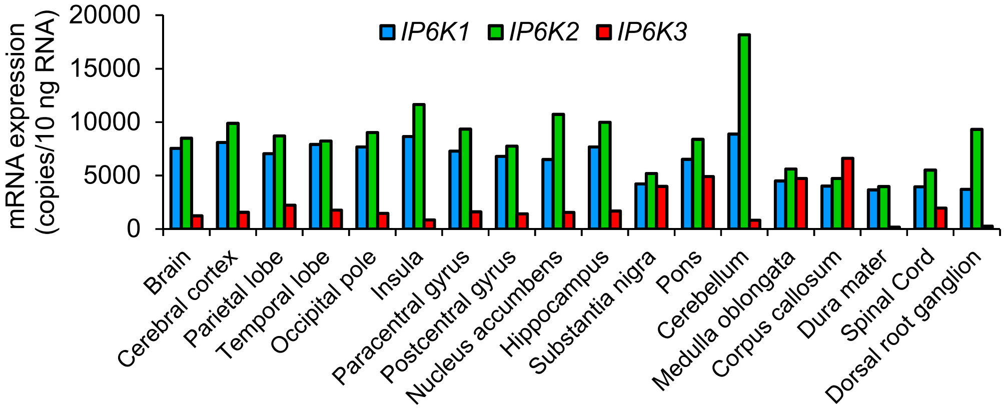

Figure S1. *IP6K3* expression in the human brain. *IP6K3* showed brain-region dependent expression. *IP6K3* mRNA levels were lower overall compared to *IP6K1* and *IP6K2*. Two technical replicates were performed per pooled human sample.

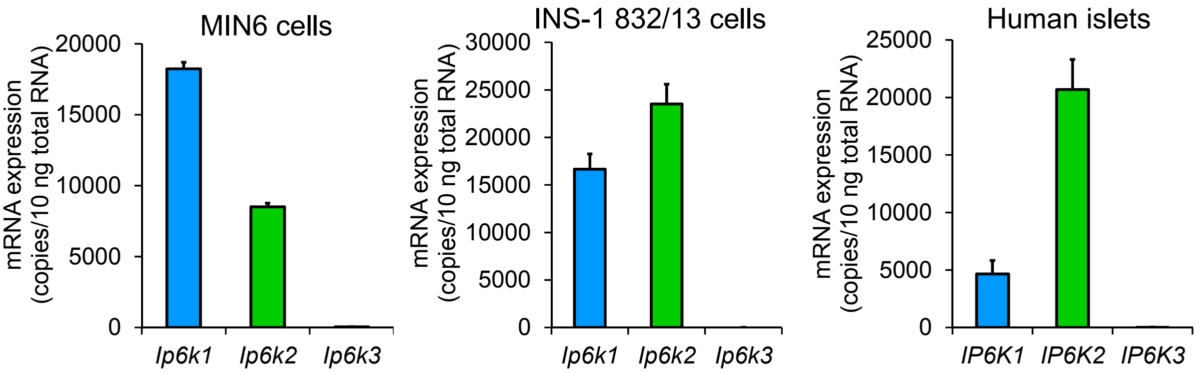

Figure S2. Rodent *Ip6k3* and human *IP6K3* are undetectable in pancreatic endocrine cells. mRNA expression in mouse MIN6, rat INS-1 832/13, and human islets. *Ip6k3* mRNA was not expressed in MIN6 and INS-1 832/13 beta cell lines. In addition, *IP6K3* mRNA was undetectable in human islets. The means ± SD of three (MIN6 and human islets) and four (INS-1) independent samples are shown. Two technical replicates were performed per sample.

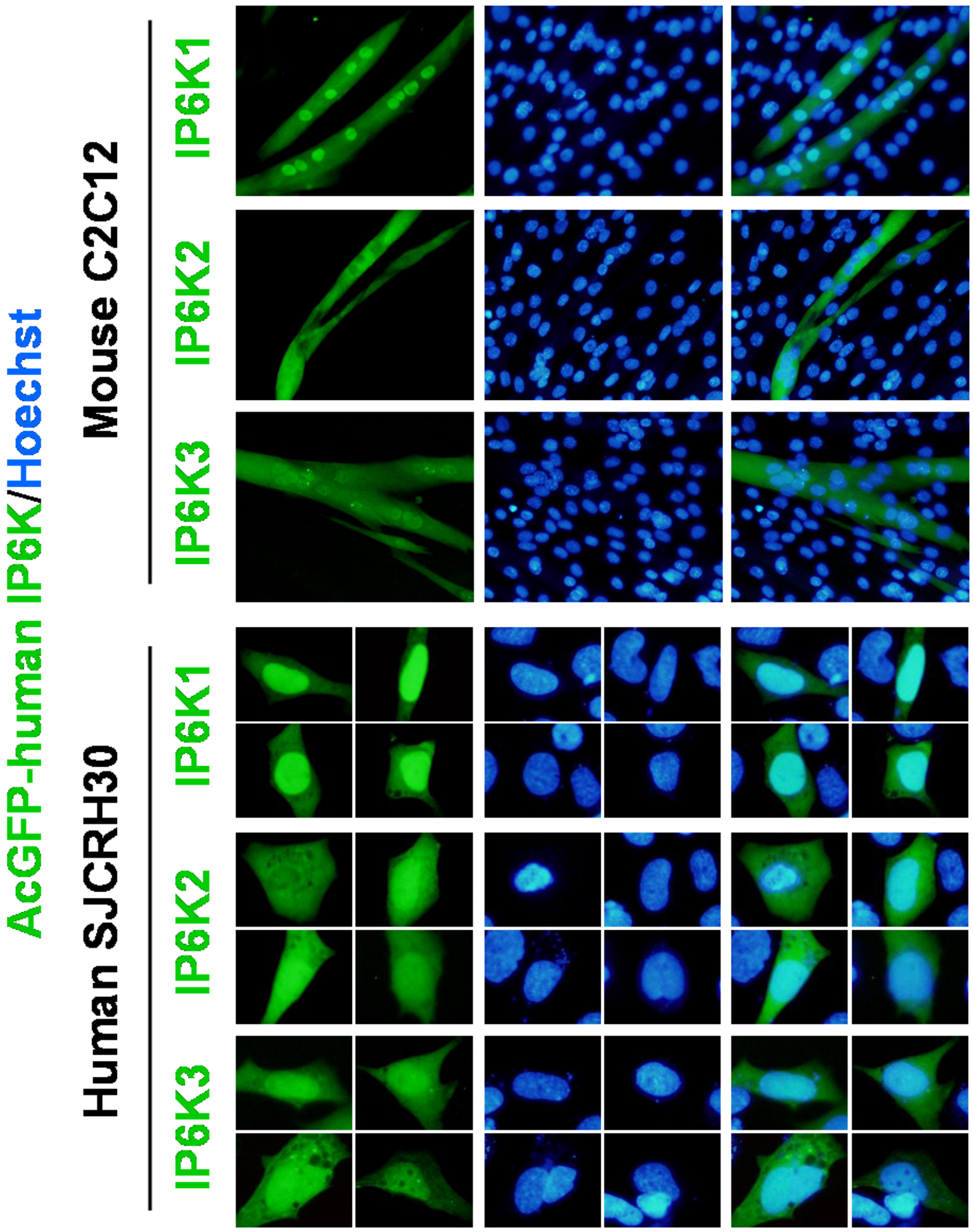

Figure S3. AcGFP-hIP6K3 fusion protein localizes to the nucleus and the cytoplasm in mouse C2C12 cells and a human SJCRH30 rhabdomyosarcoma cells. AcGFP-IP6K1, AcGFP-IP6K2, and AcGFP-IP6K3 fusion constructs were transfected into C2C12 or SJCRH30 cells. Cells were visualized with AcGFP and nuclear staining (Hoechst 33342) at 2-4 days after transfection. AcGFP-IP6K3 was localized to the nucleus and the cytosol in both cell lines. Data shown are representative images of three independent experiments. Images were obtained from a highly sensitive charge-coupled device (CCD) camera fitted to the microscope (DP73/IX71; Olympus).

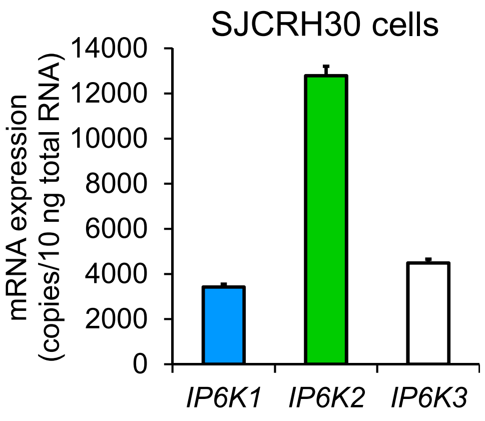

Figure S4. The human SJCRH30 rhabdomyosarcoma cell line expresses *IP6K3*

The means ± SD of four independent samples consisting of two technical replicates.

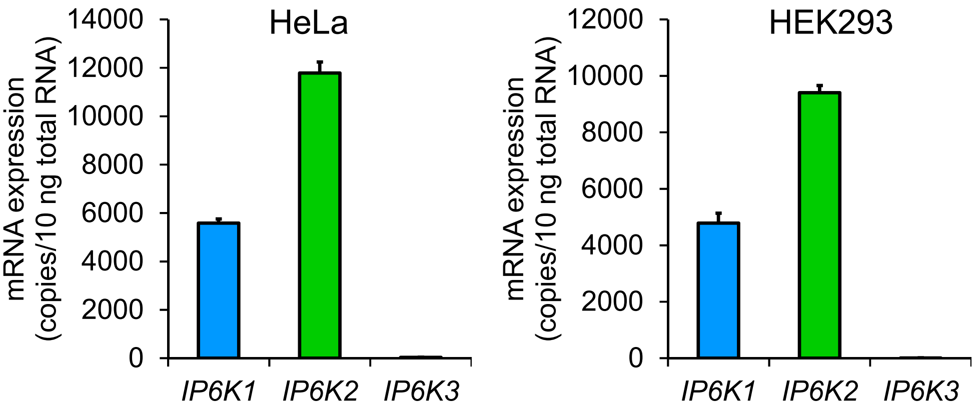

Figure S5. *IP6K1*, *IP6K2*, and *IP6K3* mRNA expression in HeLa and HEK293 cells.

IP6K3 mRNA was undetectable in human HeLa and HEK293 cell lines. The results represent the means ± SD obtained from four independent samples consisting of two technical replicates.

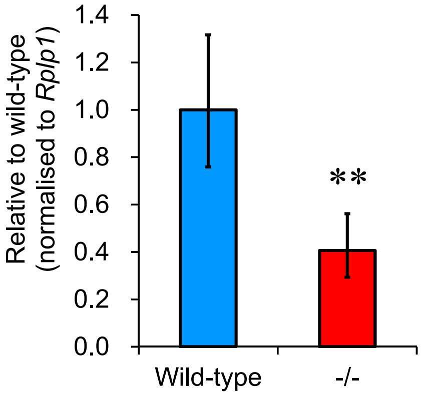

Figure S6. *Pdk4* mRNA levels in the hearts of *Ip6k3*$^{-/-}$ mice.

Pdk4 mRNA is reduced in the heart of *Ip6k3*$^{-/-}$ mice. B6;129-*Ip6k3*$^{-/-}$ and wild-type littermates at 11 weeks of age were used for the analysis . The range given for each mRNA level relative to wild-type was determined by using the comparative C_T method. The ΔC_T value is determined by subtracting the average *Rplp1* C_T value from the average *Pdk4* C_T value. The calculation of $\Delta\Delta C_T$ involves subtraction by the ΔC_T calibrator value (wild-type littermates). The data range given is determined by evaluating the expression: $2^{-\Delta\Delta CT}$ with $\Delta\Delta C_T + s$ and $\Delta\Delta C_T - s$, where s = the standard deviation of the $\Delta\Delta C_T$ value. n = 4 and 6 for wild-type and *Ip6k3*$^{-/-}$ mice. ***P*<0.01 by the Student's *t*-test.

Table. S1. Primers, probes, and standard sequences.

Sequence Name	Sequences
Hs_IP6K1-F	CAGGCAGTGGCAGTGACC
Hs_IP6K1-P	AGAAAGCCAGCCTGTCCCTTGAGACC
Hs_IP6K1-R	GGACTCTTTGCCTCCTGTGAG
Hs_IP6K1-std	CCGGTCAGGCAGTGGCAGTGACCACAAGGAGGAGAAAGCCAGCCTGTCCCTTGAGACCT CTGAGAGCTCACAGGAGGCAAAGAGTCCGAAGG
Hs_IP6K2-F	CATGCGTGCTCCGCTTCAA
Hs_IP6K2-P	AGACAACCCTGTGCAAGCCCCTGGTC
Hs_IP6K2-R	GCATCTCAGCAGGGAGGGTC
Hs_IP6K2-std	GCACTCATGCGTGCTCCGCTTCAATGAGACAACCCTGTGCAAGCCCCTGGTCCCAAGGGA ACATCAGTTCTACGAGACCCTCCCTGCTGAGATGCGCAAA
Hs_IP6K3-F	TCACCCCACAGTACAAAGGTAC
Hs_IP6K3-P	AAGACAGCACAGGCCATCTCAGCTTGG
Hs_IP6K3-R	GGCTCTCCTTCACTGGGTTG
Hs_IP6K3-std	GCGGTTCACCCCACAGTACAAAGGTACCGTCACAGTGCACCTCTGGAAAGACAGCACAGG CCATCTCAGCTTGGTTGCCAACCCAGTGAAGGAGAGCCAGGAG
Hs_IPMK-F	GGCACAGTTTTGAAACAGTTACAAC
Hs_IPMK-P	ACCTCCAAGGGGCCCAAGAGAGCTG
Hs_IPMK-R	CGTAGCTCTAGAAGAACACCATCA
Hs_IPMK-std	CAGATGGCACAGTTTTGAAACAGTTACAACCACCTCCAAGGGGCCCAAGAGAGCTGGAAT TCTATAATATGGTTTATGCTGCTGACTGTTTTGATGGTGTTCTTCTAGAGCTACGAAAAT
Hs_IPPK-F	TGGGAAGTGGAAGCAGATCAG
Hs_IPPK-P	ACTGTCCCCTTGATCTCTACTCAGGAAACA
Hs_IPPK-R	CAGCAAACTCTTCAAGGCAAAG
Hs_IPPK-std	GCAACTGGGAAGTGGAAGCAGATCAGCAAATACTGTCCCCTTGATCTCTACTCAGGAAAC AAACAGAGAATGCACTTTGCCTTGAAGAGTTTGCTGCAGGA
Hs_ITPK1-F	GTCCTACACCGTGGTCCAGA
Hs_ITPK1-P	TTCTCCGCAGGCACATCAGACCGTGA
Hs_ITPK1-R	CGTTGTGGCTGTTGAAGAAGATG
Hs_ITPK1-std	GGCGAGTCCTACACCGTGGTCCAGAGGCCCTCACTCAAGAACTTCTCCGCAGGCACATCA GACCGTGAGTCCATCTTCTTCAACAGCCACAACGTGTCA
Hs_ITPKA-F	GCGAGGACGTGGGTCAGA
Hs_ITPKA-P	ACCACTGGCAGAAGATCCGGACCATGG
Hs_ITPKA-R	CGCTTCTTGAAAGGGCTTATGA
Hs_ITPKA-std	AGCGGGCGAGGACGTGGGTCAGAAAAACCACTGGCAGAAGATCCGGACCATGGTCAATC TGCCGGTCATAAGCCCTTTCAAGAAGCGCTACG
Hs_ITPKB-F	CCTGTGTGATGGACTGCAAGA
Hs_ITPKB-P	AGCTCCTCCTCCAGGTAGGTCCTGATTC
Hs_ITPKB-R	CGCAGGCTGGGCTTCTTC
Hs_ITPKB-std	CTCGCCCTGTGTGATGGACTGCAAGATGGGAATCAGGACCTACCTGGAGGAGGAGCTCAC GAAGGCCCGGAAGAAGCCCAGCCTGCGGAAGG
Hs_ITPKC-F	CACCCTTTGTGGTCTCCTTCC
Hs_ITPKC-P	AACACTACCCTTGGGTCCAGCTTTCTGG
Hs_ITPKC-R	CCATCCTCTCCTGCCTGGAA
Hs_ITPKC-std	GTATTCACCCTTTGTGGTCTCCTTCCGAAAACACTACCCTTGGGTCCAGCTTTCTGGACAT GCTGGGAACTTCCAGGCAGGAGAGGATGGTCGGA
Hs_PPIP5K1-F	TTCCCTACGTCAAGTGAGTGAA
Hs_PPIP5K1-P	CTTGAGTAGAGTCTGCCAGCGCCACAC
Hs_PPIP5K1-R	TGCTGTGCATGGAATCAAAGAG
Hs_PPIP5K1-std	GCCCTTTCCCTACGTCAAGTGAGTGAATTCTTGAGTAGAGTCTGCCAGCGCCACACTGATG CCCAGGCACAGGCATCTGCAGCCCTCTTTGATTCCATGCACAGCAGCCAG
Hs_PPIP5K2-F	GCAGATATTGTTATCCCTCAGGAATA
Hs_PPIP5K2-P	ACTGGAGATTGCCAAAGGCTACTGTACTCC

Hs_PPIP5K2-R	AGGTCTGAGCGAATTTTTCTAACC
Hs_PPIP5K2-std	CATTAGCAGATATTGTTATCCCTCAGGAATATGGTATAACTAAAGCTGAAAAACTGGAGA TTGCCAAAGGCTACTGTACTCCTCTGGTTAGAAAAATTCGCTCAGACCTTCAGA
Hs_PDK4-F	TCGTGTATGTTCCTTCTCACCTC
Hs_PDK4-P	TGTTCAACTGTTGCCCGCATTGCATTCT
Hs_PDK4-R	GGTGTAAGGGAAGGCTGATTTTC
Hs_CKM-F	CACTGCTCCCGTGGCGAG
Hs_CKM-P	CCCGTCAGGCTGTTGAGAGCTTCCACA
Hs_CKM-R	CATGCTCTTCAGAGGGTAGTACTTC
Hs_RPLP0-F	AAACGAGTCCTGGCCTTGTCT
Hs_RPLP0-P	AGACGGATTACACCTTCCCACTTGCTGA
Hs_RPLP0-R	GCAGATGGATCAGCCAAGAAG
Mm_Ip6k1-F	TCGGTCTGGCAGTGGTAGTG
Mm_Ip6k1-P	AAGCCAGCCTGTCCTTTGAGACCTCTGA
Mm_Ip6k1-R	GTTCCACCTTTGGACTCTTTGC
Mm_Ip6k1-std	CTGCATCGGTCTGGCAGTGGTAGTGACCACAAGGAGGAGAAAGCCAGCCTGTCCTTTGAG ACCTCTGAGAGCTCCCAGGAGGCAAAGAGTCCAAAGGTGGAACTGCAC
Mm_Ip6k2-F	GCGATGATGCTTCGGAGGAA
Mm_Ip6k2-P	CCGCACACTCGAACACCGATGACCG
Mm_Ip6k2-R	CCGCCTGGTACACCTGCA
Mm_Ip6k2-std	GCATGGCGATGATGCTTCGGAGGAAAAAGCAGCTAACCAGATCCGAAAATGTCAGCAGA GCACGTCTGCGGTCATCGGTGTTCGAGTGTGCGGCATGCAGGTGTACCAGGCGGGCACT
Mm_Ip6k3-F	GACACTTCAGCAGACAACCG
Mm_Ip6k3-P	CACCTTGGCTGCACTCTCCTTCAGTGA
Mm_Ip6k3-R	GTGGCAGTCGGACCTCAG
Mm_Ip6k3-std	TGGCAGACACTTCAGCAGACAACCGGCAGCGAGAGCAGCCCCTGCCCCCTCACCCAGCTG GCTCGCTCACTGAAGGAGAGTGCAGCCAAGGTGCTCCTGAGGTCCGACTGCCACCTCAG
Mm_Pdk4-F	TGGAGTTCCATGAGAAGAGCC
Mm_Pdk4-P	AGCCCTGTCAGAGTTTGTAGACACGCT
Mm_Pdk4-R	GGGACCACATTATGATGTCTGTTTC
Mm_Rplp1-F	AGTCTACAGCATGGCTTCCG
Mm_Rplp1-P	CCGTCACCTCGTCGTCGTGCAGG
Mm_Rplp1-R	GGCATTGATCTTATCCTCCGTG

Hs, human; Mm, mouse; F, forward primer; R, reverse primer; P, probe; std, standard oligonucleotide.

All sequences are written 5' to 3'.

Table S2. TaqMan gene expression assays.

Target	TaqMan Gene Expression Assay
Ip6k1	Mm00501996_m1
Ip6k2	Mm01232057_m1
Ip6k3	Mm00557209_m1
Trim63	Mm01185221_m1
Fbxo32	Mm00499523_m1
IP6K1	Hs01051078_m1
IP6K2	Hs00382829_m1
IP6K3	Hs00369684_m1

Hs, human; Mm, mouse.
